# Characterizing stage-dependent neuromotor patterns in *Drosophila melanogaster* larvae through a graph construction approach

**DOI:** 10.3389/fnins.2025.1557624

**Published:** 2025-03-20

**Authors:** Yuri Bilk Matos, Nadezhda Velichkova, Mateo Kirchknopf Riera, Marcos Gomes Eleutério da Luz, Jimena Berni

**Affiliations:** ^1^Departamento de Física, Universidade Federal do Paraná, Curitiba, Brazil; ^2^Brighton and Sussex Medical School, University of Brighton and University of Sussex, Brighton, United Kingdom

**Keywords:** neuromotor development, locomotion, *Drosophila* larvae, calcium imaging, neuronal activity, mathematical modeling, graph approach

## Abstract

We investigated developmental changes in neuromotor activity patterns in *Drosophila melanogaster* larvae by combining calcium imaging with a novel graph-based mathematical framework. This allows to perform relevant quantitative comparisons between first (L1) and early third (L3) instar larvae. We found that L1 larvae exhibit higher frequencies of spontaneous neural activity that fail to propagate, indicating a less mature neuromotor system. In contrast, L3 larvae show efficient initiation and propagation of neural activity along the entire ventral nerve cord (VNC), resulting in longer activity chains. The time of chain propagation along the entire VNC is shorter in L1 than in L3, probably reflecting the increased length of the VNC. On the other hand, the time of peristaltic waves through the whole body during locomotion is much faster in L3 than in L1, so correlating with higher velocities and greater dispersal rates. Hence, the VNC-body interaction determines the characteristics of peristaltic waves propagation in crawling larvae. Further, asymmetrical neuronal activity, predominantly in anterior segments of L3 larvae, was associated with turning behaviors and enhanced navigation. These findings illustrate that the proposed quantitative model provides a systematic method to analyze neuromotor patterns across developmental stages, for instance, helping to uncover the maturation stages of neural circuits and their role in locomotion.

## 1 Introduction

The use of model organisms is key for the understanding of life in a broad sense, helping to unveil a large number of fundamental principles governing biological processes (Alfred and Baldwin, 2015; Ankeny and Leonelli, 2020). However, this also poses many challenges (Rine, 2014; Russell et al., 2017; Bertile et al., 2023; Myslivecek et al., 2023). Indeed, choosing a particular species as a “generalist case” might raise relevant questions such as its representativeness of holobionts, its reaching as a genetic prototype, and the universality of its translational physiology, to list just a few issues. Moreover, to adequately serve as a model, one needs a proper, quoting (Leonelli, 2013): “standardization of the organism in question and the accumulation of knowledge and resources on the organism on a large scale.”

Although general methods have been proposed to deal with non-model species (Bertile et al., 2023; Williams et al., 2011), to work with certain particular organisms is still the main trend, whose usual choices tend to be bacteria, yeast, worms, mice and flies (Perillo, 2017). In particular, *Drosophila melanogaster*, commonly known as the fruit fly, has long been central in developmental biology studies (Yamaguchi and Yoshida, 2018; Lewis, 2004). For example, its relatively simple genetic makeup and short generation time makes the *Drosophila* ideal for the investigation of a wide range of processes, e.g., related to genetics, ontogeny, learning, development, aging, etc. (Stearns et al., 2000; Phillips et al., 2022). Furthermore, *Drosophila* nervous system is complex enough to share many structural similarities with more intricate nervous systems, including human, yet simple enough to be feasibly modeled.

Among the various life stages of the *Drosophila*, the larval one represents a critical period of growth and transformation, punctuated by changes in physiology, morphology and behavior (Bate and Arias, 1993). This is precisely why such stage has been proposed as an important instance to survey the relationship between brain progressive development and the associated behaviors (Gerber and Stocker, 2007). Thus, substantial efforts have been made toward mapping the *Drosophila melanogaster* neural system (Pfeiffer et al., 2008; Zheng et al., 2018; Naddaf, 2023). Characterizing the neuromuscular network has been particularly fruitful, as motor activity translates directly into observable and measurable actions. Techniques such as calcium (Ca) imaging have been instrumental in visually identifying neural clusters of motor neuron activity associated with muscular contractions in the body segments (reviewed by Kohsaka et al., 2017 and Kohsaka, 2023). Also, analyses have indicated potential similarities between motifs of neural activity and segmental contractions in *D. melanogaster* larvae (Pulver et al., 2015).

Nonetheless, the depiction of the resulting data are usually aimed to disclose the activity propagation patterns in the ventral nerve cord (VNC), e.g., forward and backward wave fronts, not addressing a proper quantification of the underlying dynamics. Actually, despite all the progress, it is still not totally clear how these nervous system patterns control alternations and duration of the crawls and turns (the elementary movement steps given rise to the locomotion trajectories). In fact, such control—conceivably functioning at different hierarchical (and time-scale) levels (Anteneodo and Da Luz, 2010) of the individual body structural organization—needs to be mediated by feedbacks with the environment. But this kind of trade-off interaction is also not fully known (Koyama et al., 2020; Davies et al., 2021). The bottom line is that contrary to the rapid advances in experimental techniques (Davis, 2023), the aforementioned knowledge gaps are, at least partially, due to an insufficiency of more appropriate mathematical frameworks. It is conceivable that more robust analysis and simulation methods could describe the brain patterns involved, allowing to link them to emergent motor responses and actions. As recently demonstrated in a breakthrough work (Shiu et al., 2024), theoretical tools are paramount to comprehend the sensorial processes in the *Drosophila* brain.

Given the above, as a novel protocol to delineate and typify the neural-motor activity patterns of *Drosophila* larvae, we propose a graph theory approach to characterize the structural patterns of neuronal activity waves. It allows to quantitatively assess asymmetry and propagation patterns across the VNC. This is achieved by processing Ca imaging data such that each burst of neuronal signal corresponds to an “event,” represented by a graph node ascribed to the VNC activated region. As activity propagates across different VNC regions, the associated activation flow is represented by directed graph edges. This ensures that the geometrical structure of the resulting graph mirrors the physical arrangement of the triggered VNC regions.

**Figure 1:**
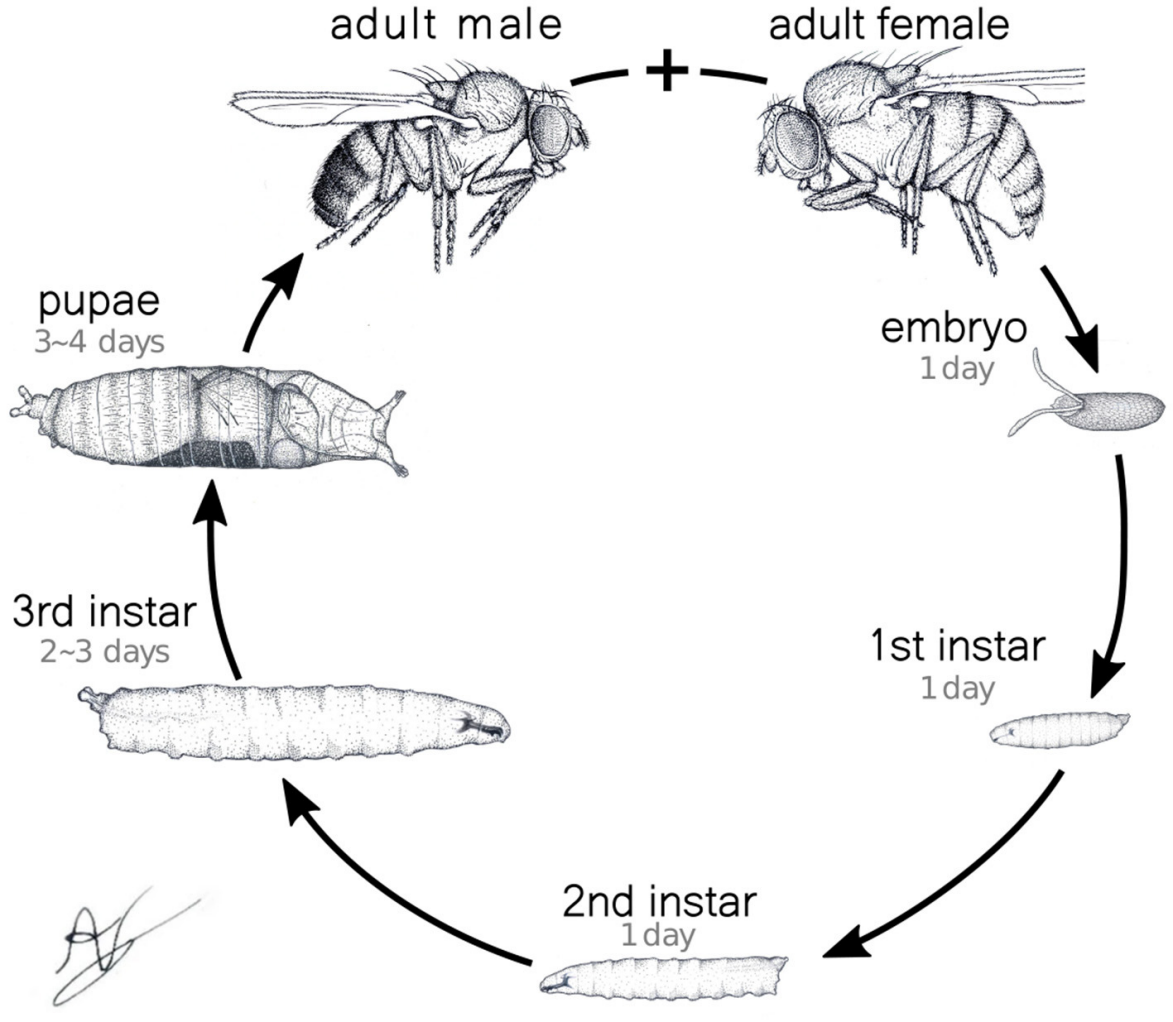
*Drosophila* full life cycle. In particular, the three larval phases, the instars, are explicitly shown. The characteristics times of the transitions between different developmental stages are also indicated.

Based on such mathematical construction, among other aspects one should be able to compare activity behavior in larvae across different developmental stages, thereby describing the evolution of motifs of neuronal activity underlying movement. To demonstrate that this is indeed the case, we have performed many controlled measurements (details in the following) explicitly considering the larvae's specific developmental phases. After emerging from the egg, the *Drosophila* undergoes three growth stages, the instars (see Figure 1). Each stage is separated from the previous one by a cuticle molt to accommodate their remarkable size increase. Throughout the first, second, and the beginning of the third instars, the larvae display foraging behavior characterized by continuous feeding and a permanent search for food. This feeding frenzy is vital to sustain the mentioned rapid growth and to guarantee enough energy reserves for surviving metamorphosis (Tennessen and Thummel, 2011). So, the significance of larval movement extends beyond mere locomotion. In consequence, the regulation of foraging patterns has far-reaching implications for ecological interactions, sensory perception, and ultimately, the fitness of the organism. Naturally, all these processes are driven by the patterns of neuromotor activity in the animal's VNC.

In the experiments, we recorded detailed neuromotor activity of the first (L1) and third (L3) larvae instars (with an one stage lag chosen so to warranty a clear developmental maturation between phases) and applied the proposed graph-based scheme to investigate the structural characteristics of neuronal activity underlying specific behavioral outputs, such as turning and crawling. Additionally, we tracked larval trajectories in a custom-designed measurement arena to examine dispersal behavior (Almeida-Carvalho et al., 2017) and evaluated how differences in neuromotor coordination influence movement dynamics. For this type of locomotion data, besides L1 and L3, we also considered the second (L2) instar. By comparing the neural structures across the stages, our treated data clearly established that activity propagation and generation in L1 larvae were less coordinated compared to L3, reflecting the immaturity of the former neural networks. More importantly, these differences could be concretely quantified by relating distinct “motifs” to the graphs representing the neural firing circuits. Indeed, their types, frequency of occurrence, correlation and temporal sequence indicated the basic motion steps being executed and how efficiently and synchronized the neural signaling induced movement and spatial dispersion.

The structure of our works is as follows. In section 2 we first describe the experimental procedures, including the rearing of *Drosophila* larvae, the recording of their locomotion trajectories and the Ca imaging techniques employed to monitor neuromotor activity. Second, we introduce our mathematical modeling approach based on graph theory and motifs identification, which is used to analyse and quantify the propagation patterns of neuronal activity within the VNC. Section 3 presents our key findings, highlighting the differences in spontaneous activity generation, activity propagation efficiency, and symmetry patterns between first instar (L1) and early third instar (L3) larvae. Finally, section 4 summarizes the implications of our study, emphasizing the developmental progression of neuromotor coordination and suggesting potential directions for future research.

## 2 Methods

### 2.1 *Drosophila* larvae rearing

Larvae neuronal activity was assessed at two distinct stages of their development, first instar (L1) and early third instar (L3). For the recording of locomotion trajectories we also assessed second instar (L2). To establish the experimental sample, 30 female and 20 male adult flies of the wild type OregonR strain (OrR) were placed in a laying pot and provided with a standard corn media enriched with yeast paste Petri dish to lay eggs. The eggs were collected for 2 h and incubated either for 24 h (first instar; L1), 48 h (second instar; L2) or 74 h (early third instar; L3) at 25°C under a 12-h light-dark cycle. Three sets of larvae were produced for each developmental stage. This controlled setup ensures synchronized development and consistent conditions for subsequent analyses of locomotion and trajectory patterns.

### 2.2 Recording locomotion trajectories

We conducted observations of larval exploratory behavior in controlled environments under minimal external stimuli. The recordings were conducted in the dark apart from infrared light that the larvae can not see, maintaining a consistent temperature of 25°C. Each experimental trial spanned 60 min, during which the larvae were tracked within a 240 × 240 mm^2^ arena. The arena surface was prepared with a 0.4% agar-based coating, 2 mm in thickness.

In each trial, a group of 10 larvae of similar size was put to roam at the arena, and each developmental stage (L1 and L3) was tested with three repetitions (summing up 30 larvae per stage). Their movements were recorded through a frustrated total internal reflection (FTIR)-based imaging technique (Risse et al., 2013) using a Basler acA2040-180 km CMOS camera set at a resolution of 2,048 × 2,048 pixels. The recording was performed at two frames per second, optimized for accurate representation of forward displacements and actual pause-turn events, while minimizing the inclusion of “flickering” movements (often associated with peristaltic contractions). To enhance the observations precision, an advanced imaging setup was employed, featuring a 16 mm KOWA IJM3sHC.SW VIS-NIR lens and a 825 nm high-performance longpass filter (Schneider, IF-093). For the recording of the first instar larvae L1, an additional 2 × amplification lens was used.

**Figure 2:**
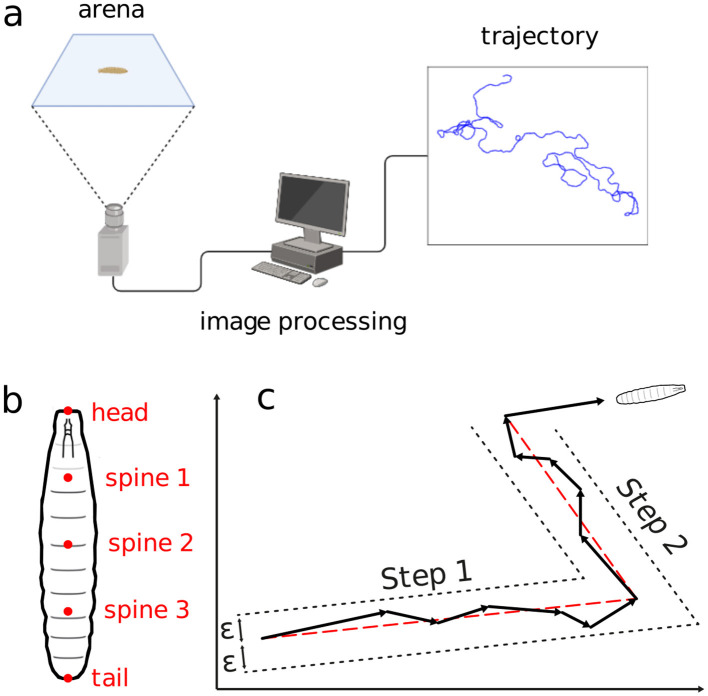
Schematics of the movement experiment and type of data gathered. **(A)** Arena, image processing setup, locomotion trajectory. **(B)** Positional measurement points along the larvae's body. **(C)** Displacement aggregation method to generate random walk steps.

The images were then processed with the FIM-track software (Risse et al., 2017), yielding positional time series for each of the larva's trajectories, see the schematics in Figure 2A. To make the trajectories as free from the other organisms as possible, a tracking was always interrupted upon any collision between two distinct larvae. So, two new trajectories were assumed as the interacting larvae would get enough away apart. Similarly procedure was considered when the larvae reached the arena's edge. Figure 2A illustrates the experimental setup.

### 2.3 Recording locomotion wavelength

To evaluate the number of body segments along which a peristaltic wave of muscle contraction progresses (here operationally defined as “wavelength”), we recorded movies in L1 (15 individuals) and L3 (10 individuals). Larvae were transferred to a 5 cm petri dish coated with 0.6 ml of 0.9% agarose. The plate was inverted to view the denticle bands and 2 min movies were captured at 30 fps with a ximea MQ013CG-ON camera mounted on a Leica M420 microscope at 25 × (L1) and 6 × (L3) magnification. Larvae only executed forward peristaltic wave and their progression was determined by the number of segments contracting as revealed by denticle belts movements that are located on the boundary of each segment and clearly visible from A8 to A1. The movement of the thoracic segments T3, T2 and T1 was evaluated by the movement of A1 and the front of the animal, thus representing three segments. The events were quantified with the open source software VCode 1.2.1 (http://social.cs.uiuc.edu/projects/vcode.html).

### 2.4 Descriptive statistics of larval trajectory

Positional data, i.e., (*x, y*) coordinates of an individual larvae trajectory *i* in each video frame *j* (so a characterization of time), is represented by the vector


(1) $$\begin{array}{l} \vec{R_{i}}\left(t_{j}\right)=\left(x_{i}\left(t_{j}\right),y_{i}\left(t_{j}\right)\right), \end{array}$$

where (*x*_*i*_(*t*_*j*_), *y*_*i*_(*t*_*j*_)) represents the centroid coordinates of the larvae at time *t*_*j*_ = *jΔt*. Here, Δ*t* = 0.5 s is the temporal resolution, *j* = 1, 2, …, *N* the frame index and *N* = 6000 the number of frames per experiment. Measurements include the positions of the head, tail, and three spine points for each larva in every frame (Figure 2B). The body length *L*_*i*_(*t*_*j*_) at *t*_*j*_ is computed as the sum of distances between consecutive body points; an average 〈*L*_*i*_〉 is calculated for each trajectory.

Trajectories are segmented into quasi-linear portions, defined as stretches of more or less straight displacements, delimited by the organism directional changes. The movement in a sequence of frames are aggregated into a single step if the intermediate frame positions all fall within a distance ϵ from the line connecting the first and last frame positions (Turchin, 1998)—refer to in Figure 2C. The distance ϵ is defined as half the average body length at each trajectory, plus half the standard deviation, namely,


(2) $$\begin{array}{l} \epsilon =\langle L_{i}\rangle /2+\sqrt{\langle L_{i}^{2}\rangle /2}. \end{array}$$

The aggregation of frame-movements into steps is performed with a Ramer-Douglas-Peucker algorithm so to find the combination that best represents the trajectory (Wosniack et al., 2022; Ramer, 1972). The velocity *V*_*k*_ is defined in terms of the average velocity across each linear step


(3) $$\begin{array}{l} V_{k}=\ell_{k}/\Delta T_{k}, \end{array}$$

where ℓ_*k*_ is the linear displacement of the step *k* and Δ*T*_*k*_ its duration. *V*_*k*_ is then normalized in relation to body-size by dividing it by 〈*L*_*i*_〉.

To quantify the degree of dispersion of the trajectory *i* (starting at *t*_*a*_ and ending at *t*_*b*_), we consider the Mean Square Displacement (MSD) over a time window τ, MSD_*i*_(τ), computed by averaging all possible displacements of duration τ along *i*


(4) $$\begin{array}{l} {\text{MSD}}_{i}\left(\tau \right)=\frac{1}{n}\overset{t=t_{b}-\tau }{\underset{t=t_{a}}{\sum }}\left[{\left(x_{i}\left(t+\tau \right)-x_{i}\left(t\right)\right)}^{2}+{\left(y_{i}\left(t+\tau \right)-y_{i}\left(t\right)\right)}^{2}\right]. \end{array}$$

Here, *n* is the number of displacements counted in each *i*. The overall MSD(τ) for the entire set of trajectories follows by averaging the MSD_*i*_(τ)'s, normalized by their respective mean body length (so to yield values in units of individuals sizes).

### 2.5 Calcium imaging

**Figure 3:**
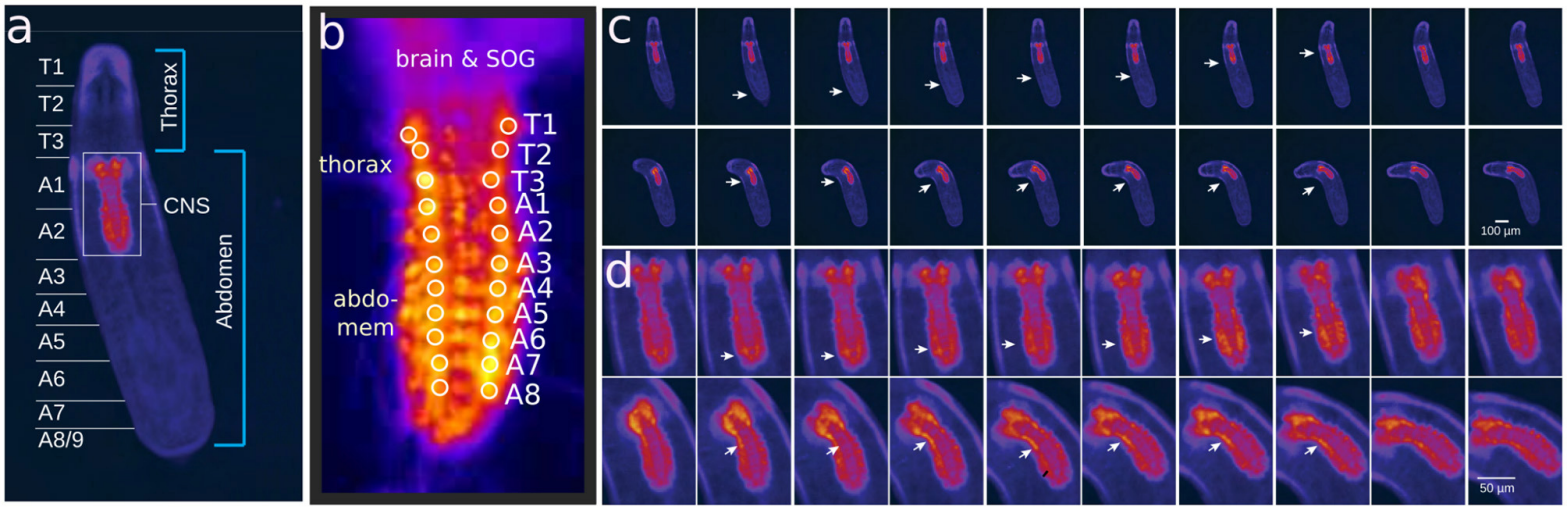
Calcium imaging technique and representative results. **(A)** *Drosophila*'s body with indicated morphological structures. **(B)** The VNC and corresponding ROIs located in each segment. Correlation between bodily movements **(C)** and VNC activity **(D)**.

For calcium imaging of the central nervous system (CNS), whose location in the individuals body is indicated in Figure 3A, the GAL4-UAS system was employed to activate the calcium indicator GCaMP3 (Tian et al., 2009). Specifically, the OK371-GAL4 driver (Mahr and Aberle, 2006) was used for expression in motor neurons and R36G02-GAL4 for A27h (Fushiki et al., 2016).

Thus, individual first and third instar larvae were dissected using hypodermic needles. The central nervous system (CNS), including the brain, subesophageal ganglion (SOG), and ventral nerve cord (VNC), was delicately separated from the larval body wall (Figures 3A, B). It was then mounted dorsal side up on a cover slide coated with 0.1% poly-L-Lysine (Sigma P8920). To ensure stability, recordings commenced at least 5 min post-dissection. Throughout the dissection and subsequent Ca imaging experiments, the CNS was bathed in a physiological saline solution containing (in mM) 135 NaCl, 5 KCl, 2 CaCl_2_, 4 MgCl_2_, 5 TES and 36 sucrose.

For live imaging of the isolated CNS, wide-field epifluorescence microscopy was employed. We used a cool LED simply better control PE-300white at 1% intensity for uniform illumination at 488 nm, with imaging conducted using an Olympus BX50WI compound microscope (Olympus, Center Valley, PA). The emitted light was filtered through GFP emission filters before being captured by an Hamamatsu Orca Flash 4.0 (Hamamatsu Photonics K.K). Image capture rates were set at 5 Hz using HCImage software, maintaining constant gain settings. We analyzed fluorescence values from regions of interest (ROIs) in thoracic (T1–T3) and abdominal (A1–A8) ganglia (Figure 3B). The optical intensity *I*_*i*_ = Δ*f*/*f* were smoothed with a moving average in the range of 3 s, the asymmetric least squares method (Peng et al., 2010) was used to correct the signal baseline, and the data normalized to put the signals within a quantitative range from 0 to 1, where 0 shows minimum activity and 1 maximum activity. Typical results are presented in Figures 3C, D.

### 2.6 Mathematical modeling and a novel graph characterization

**Figure 4:**
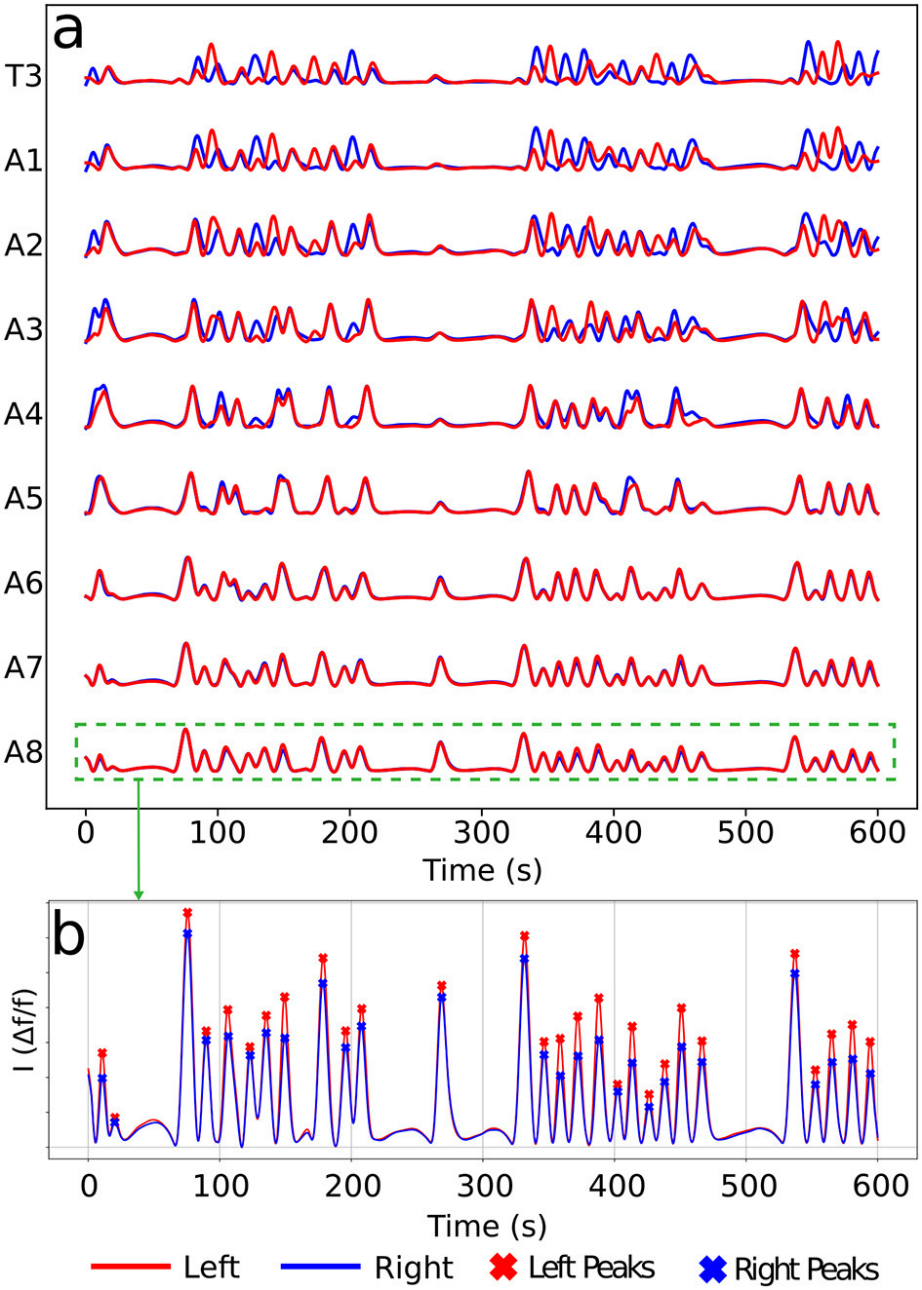
Typical calcium imaging signals. **(A)** A set of neural activity pulses for left and right A1–A8 and T3 (T1 and T2 are very similar, so not shown). **(B)** Peak patterns along a pulse of a given ROI (here A8). Each peak corresponds to a neuromotor activity event in a ROI. For the later graph construction, vertices will be associated to left (red) and right (blue) peaks as the present ones.

**Figure 5:**
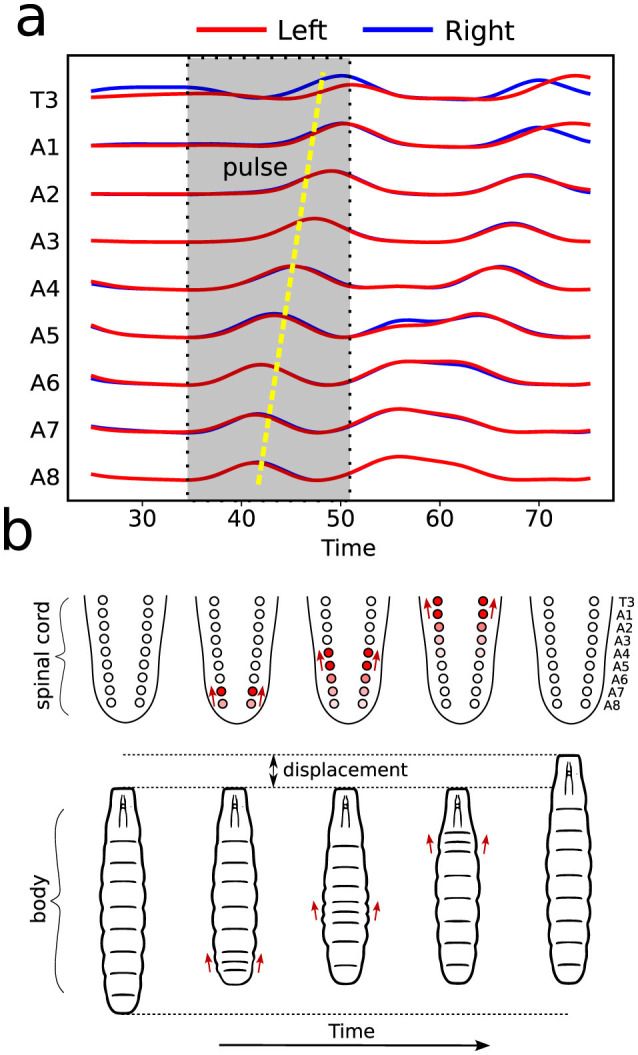
Illustration of neural and muscular activities correlation. **(A)** Activity pulses traces taking place in distinct ROIs, as measured by calcium imaging. As indicated, the emerging pattern corresponds to a forward wave (from A8 onward). **(B)** The dynamics of VNC—equivalent to vertebrates spinal cord—wave behavior and the resulting segment contractions during a forward crawling wave.

Calcium imaging measurements lead to curves as shown in Figure 4A, each representing neural activity within one neuronal ROI and ranging from A1 to T3 on both the left and right sides (Figure 4B). Given that crawling behavior emerges from a coordinated pattern of peristaltic segment contractions (Heckscher et al., 2012; Gjorgjieva et al., 2013), we should expect the ganglia neuromotor activity to correlate with muscular contractions in the corresponding body segments, as shown in Figures 3C, D. In this way, calcium imaging of the larval VNC reveals a pulse propagation along ganglia, similar to peristaltic contractions (Figure 5).

These results clearly indicate that clusters of neural activation—whose local intensities variation can be monitored from the images—are essential for producing functional locomotor behavior. Nevertheless, the study of neuronal propagation across ganglia tends to be mostly descriptive. Indeed, it expresses bursts as “waves,” but typified just in terms of either forward (toward the anterior neural system part, i.e., A8 → T1) or backward (toward the posterior neural system part, i.e., T1 → A8) propagation. Further classifying the stimulations as either symmetrical, on both the left and right sides, or asymmetrical, on one side only.

It is well known that the proper characterization of an evolving network structure representing a system dynamics is able to uncover many of its properties (Albert and Barabási, 2000; Wang et al., 2015; Perra et al., 2012; Zhang et al., 2021). Hence, based on the type of data we typically acquire from calcium imaging experiments, we have developed a mathematical framework, a graph-based model, to quantitatively describe the mentioned neural processes. The primary objective of such an approach is to prevail over the limitations of the previously mentioned qualitative analyses. Concretely, to identify different kinds of VNC activities and even ascertain how the movement patterns may be regulated by left/right ganglia sides switches. Hence, rather than just classifying a signal as forward or backward, the generated graphs should also single out the local traits of the propagating waves (in each ROI). Additionally, they should unveil symmetrical features, such as intensity and phase differences, something not possible by dividing the images into two halves and treating them as simplified binary data.

So, the key presumption is to be able to map each set of neural activity, represented by a certain collection of signals as in Figure 4A, to a graph. Naturally, the stemmed graphs are only a partial representation of the full process, but as we demonstrate in the following sections, they already yield significant information about the actual phenomenology. Consecutive activities result in distinct graph architectures, generating successive motifs. The investigation of the sequential engendering of these motifs and their correlation and frequency should give relevant quantifiable information about the neurons signals propagation and how they might related to motor behavior.

**Figure 6:**
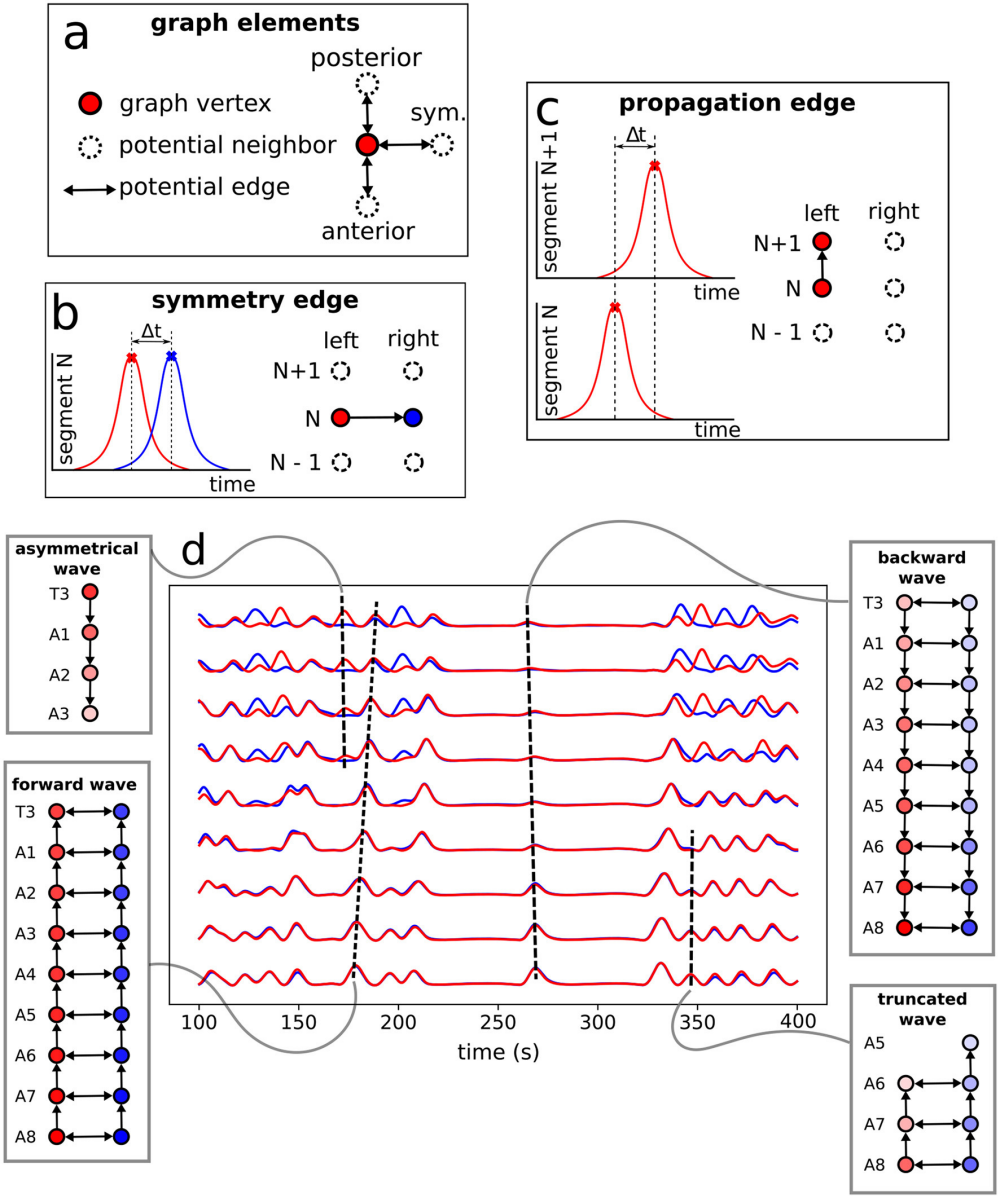
Neural activity signals and graph representation. **(A)** Basic elements (vertices and edges) used to portray neural events as a graph. A directed edge linking two vertices in adjacent ROIs (refer also to Figure 4) is established when both events, firing peaks, are within a time range Δ*t* ≤ τ = 3s —determined from data, as discussed at the end of Section 2.6. **(B)** The creation of a symmetry edge, joining vertices in different sides. **(C)** The creation of a propagation edge, joining vertices in different segments. **(D)** Activity signals in each neural cluster with the constructed motifs (sub-graphs of the full *G*) representing the associated pulses.

The protocol to construct a graph *j* is implemented as the following (refer to Figure 6 as a schematic guide). We assume a specific collection (labeled *j*) of signals, as those displayed in Figure 4A (for how to specify the different *j*'s, see below). Then, we first identify the different peaks *i* (*i* = 1, 2, 3, …) (Figure 4B) for each activity curve from every ROI in *j*. Notice the peaks have two fundamental properties: (a) intensity *I*_*i*_ = Δ*f*/*f* (more details in Section 2.5), which can be normalized between 0 and 1, where 0 and 1 indicate, respectively, the baseline and the overall maximum along the whole activity curve; and (b) occurrence time *t*_*i*_, i.e., the instant the peak *i* reaches the intensity *I*_*i*_. In this way, each *i* corresponds to an activity event $a_{i}^{\left(j\right)}\left(s_{i},l_{i},I_{i},t_{i}\right)$ of *j*, where *s*_*i*_ is the segment (A1–A8, T1–T3), *l*_*i*_ is the side (left or right), *I*_*i*_ is the normalized intensity and *t*_*i*_ is the peak time instant. Second, vertices (or nodes) *v*_*i*_ are assigned to all these peaks *i* in the collection *j*, forming the set {*v*}_*j*_ (Figure 6A). Last third, we represent the *j* sequential firing patterns across ROIs—essentially an activity chain—by means of a directed graph $G_{j}\left(v,\vec{e}\right)$. For so, we consider the vertices set {*v*}_*j*_, connecting them through a set of directed edges ${\left\{\vec{e}\right\}}_{j}$, classified into two kinds, symmetry and propagation (Figures 6A–C). The construction of ${\left\{\vec{e}\right\}}_{j}$ obeys the straightforward rule: if two adjacent ROIs are on the same segment (side), but correspond to different sides (segments), and their activity peaks, represented by vertices $v_{i^{\prime \prime }}$ and $v_{i^{\prime }}$ taking place at $t_{i^{\prime \prime }}$ and $t_{i^{\prime }}$, are such that $0<\Delta t=t_{i^{\prime \prime }}-t_{i^{\prime }}\leq \tau$, a symmetry (propagation) directed edge ${\vec{e}}_{i^{\prime }i^{\prime \prime }}$ from $v_{i^{\prime }}$ to $v_{i^{\prime \prime }}$ is established, refer to (Figures 6B, C).

**Figure 7:**
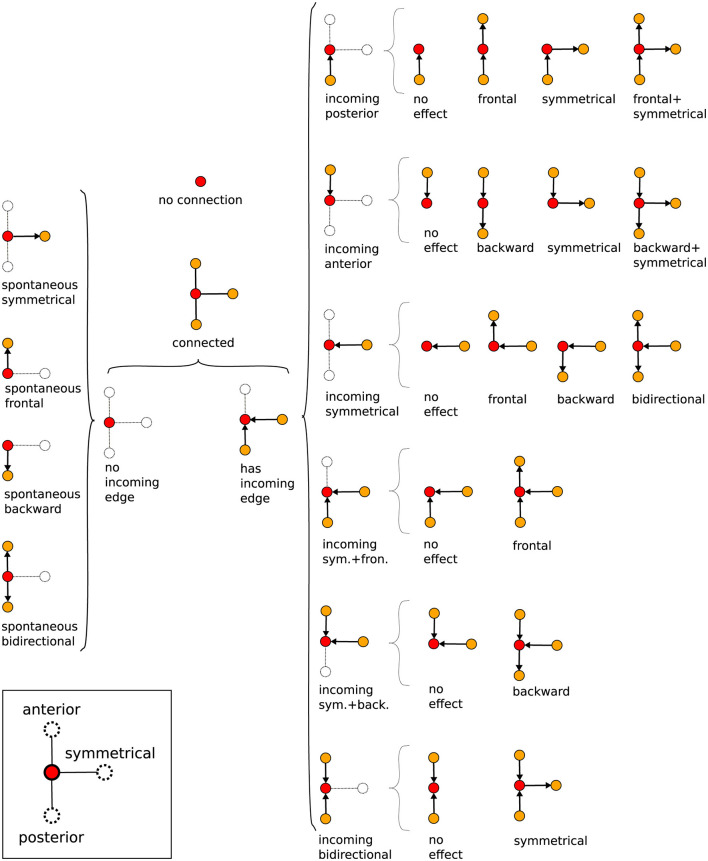
Tree of possible propagation motifs resulting from the graph construction and their nomenclature (depending on the directional flow). The motifs, basic small sub-graphs, have a central reference vertex, red dot, which then may or may not be connected to other vertices, yellow dots, by oriented edges.

Thus, *G*_*j*_ gives a general picture of the full neural activity instance *j*, with the vertices of *G*_*j*_ associated to the different events $a_{i}^{\left(j\right)}$ and the directed edges of *G*_*j*_ indicating their temporal connection. In fact, ${\left\{\vec{e}\right\}}_{j}$ is a direct portrayal of the neuronal activity propagation, as illustrated in Figure 6D. Consequently, typical neural activity patterns (for example, the forward wave in Figure 6D) are represented as motifs, i.e., simple sub-graphs of *G*_*j*_, see Figure 7. By investigating successive graphs, …, *G*_*j*−1_, *G*_*j*_, *G*_*j*+1_, …, it is possible to infer statistical properties of activity propagation by comparing ordering, correlation and relative frequencies of such motifs.

We finally mention how to identify different *G*_*j*_'s. Typically, the span of a full calcium imaging experimental run is of about *T*_run_ = 10 min, along which one clearly observes intense neural firing activity in the brain. But such long series is composed by many bursts, namely, chains of activation separated by short temporal intervals. Each one of them—e.g., the collection of signals in Figure 4A—constitutes thus a specific *j* and accordingly a graph *G*_*j*_. Taking the whole runs and computing the average interval between successive (in time) peaks, we found $\bar{\Delta t}=0.618$ s and σ_Δ*t*_ = 0.427 s for L1 and $\bar{\Delta t}=0.755$ s and σ_Δ*t*_ = 0.234 s for L3, hence in both cases with a fair effective ${\Delta t}_{\text{eff}}=\bar{\Delta t}+\sigma_{\Delta t}\approx 1$ s. In this way, for the above threshold we set τ = 3Δ*t*_eff_, establishing the maximum delay between two connected peaks of τ = 3 s. Importantly, in most cases 3 s fits with the normal times separating empirically clear distinct bursts (identified by direct visual inspection), despite the fact that the above factor of 3 was chosen somewhat arbitrarily. We define as *J* the total number of graphs constructed from an experimental series. Among all the *J* graphs, we define *J*_1_ as the number of trivial ones, i.e., those having just a single vertex.

## 3 Results

Based on the novel protocol of analysis proposed (relying on a graph construction), next we demonstrate its usefulness in characterizing and interpreting the experimental data obtained.

### 3.1 Spontaneous activity

We shall name “spontaneous” activity events those whose associated vertices have no incoming edges, refer to Figure 7. So, they are not triggered (at least within a time interval Δ*t*) by spikes in neighboring ROIs. On the contrary, these events are the ones that eventually can initiate waves of neural activity, meaning they are the starting vertices of any graph *G*_*j*_ (see the discussion at the end of Section 2.6).

The L1 and L3 larvae exhibit very similar main frequencies of spontaneous activity (Table 1), calculated as the average of *J*/*T*_run_ (Section 2.6). However, spontaneous events in L1 larvae are slightly more prone to not triggering a chain of neural activity (27.50%) than in L3 (20.00%), generating trivial graphs with a unique vertex. Statistical analysis supports this observation, as the metric *nodes_no_incoming_no_outgoing_edges* was significantly higher in L1 than in L3 (*t* = 3.13, *P* = 0.012), indicating a greater occurrence of trivial graphs in L1. Meanwhile, the metric *nodes_no_incoming_edges* showed no statistically significant difference between groups (*t* = 1.67, *P* = 0.129), suggesting that while L1 larvae exhibit a higher mean, the difference is not substantial between developmental stages. This higher failure rate results in a larger value of *J*_1_/*J* for L1. On the other hand, among the spontaneous activity events that successfully initiate an activity propagation (AP); for L1 we find a fairly equal distribution between forward and backward waves (~35%) and just a little lower percentage of both directions waves (~30%); but for L3 close to half (~45%) of the waves are forward, whereas 35% are backward and only 20% are both directions waves. But despite the mentioned contrasts, the standard deviations of each one of these proportions are similar for the L1 and L3 larvae, details in Table 1.

**Table 1:** Frequency of the spontaneous events (SE) among all events (*J*/*T*_*run*_) and relative percentage of their associated activities, averaged over each larvae population.

**AP type from SE**	**Mean L1 (SD)**	**Mean L3 (SD)**	**95% CI L1**	**95% CI L3**
Frequency of SE in Hz	0.117 (0.040)	0.118 (0.036)	[0.088, 0.146]	[0.092, 0.144]
% of SE given rise to AP	72.50 (6.72) %	80.00 (10.47) %	[67.693, 77.307]	[72.510, 87.490]
% of forward AP	35.48 (13.25) %	44.42 (13.59) %	[26.002, 44.958]	[34.698, 54.142]
% of backward AP	34.51 (11.31) %	34.62 (11.97) %	[26.419, 42.601]	[26.057, 43.183]
% of both directions AP	30.01 (11.35) %	20.96 (8.19) %	[21.891, 38.129]	[15.101, 26.819]

SD, standard deviation; CI, confidence interval; AP, for activity propagation.

**Figure 8:**
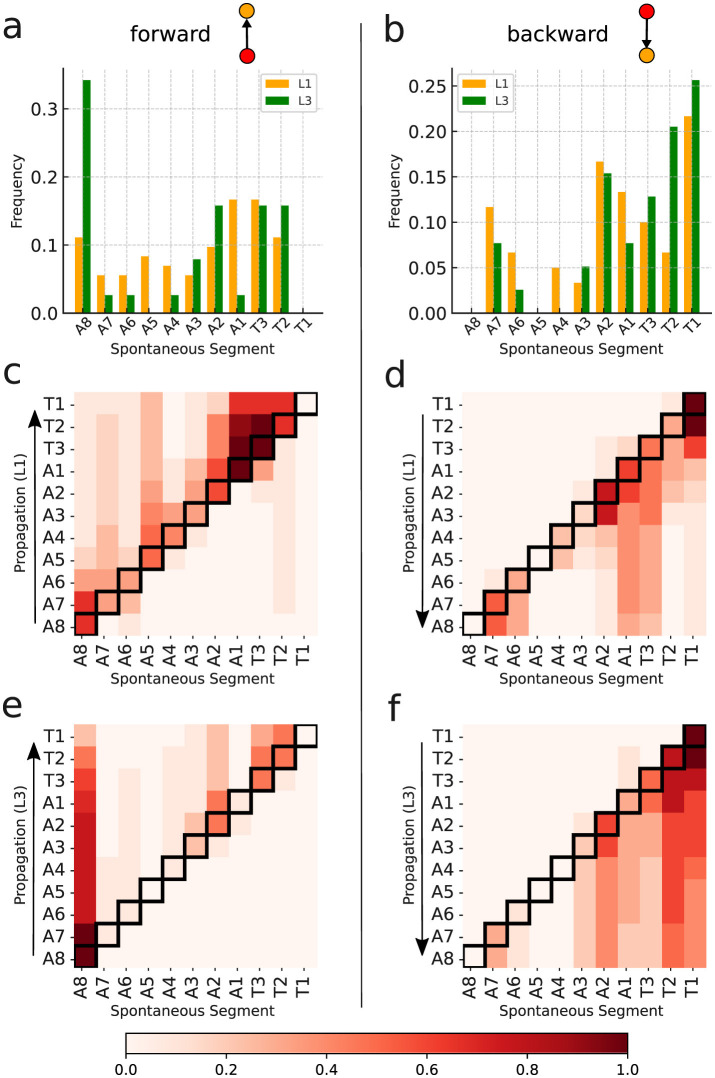
Spontaneous activity per segment of L1 and L3 larvae. **(A, B)** The distributions for segments initiating forward and backward waves (the elementary motifs in the inset). **(C–F)** Heat maps in which the horizontal axes marks the origin of the activity chains, thus corresponding to the locations of the spontaneous segments (or vertices). The propagation occurs along each row (as shown by arrows). The color intensity indicates how frequently a given segment contributes to the associated propagation chain.

Interestingly, the distributions of spontaneous activity among segments display certain distinctions for L1 and L3. Indeed, as the forward propagation, the L3 larvae present a clear dominating peak at A8, absent for L1 (Figure 8A). However, for backward propagation, although the highest peak for L3 is at T1, we still have other important peaks, notably at T2. Such trend for backward propagation, in the sense there is not an unique very pronounced peak, is the same for L1 (Figure 8B). But notice that for L1, the second most frequent activity is not at T2, but instead at A2. The prominence of A8 and T1 corresponds to the expected initiation points of behavioral contraction waves (tail for forward, head for backward). To better characterize these dissimilarities between L1 and L3, we used the Kolmogorov–Smirnov (KS) test (Dodge, 2008), which evaluates whether or not two distributions differ significantly by comparing their cumulative distribution functions (CDF). For the forward propagations per segment of L1 and L3, the KS statistic resulted in 0.545, with a *P*-value of 0.07. This near-threshold *P*-value suggests that L1 and L3 distributions are likely governed by distinct processes, albeit with a limited statistical certainty. Conversely, for backward propagations, the KS statistic was 0.273, with a *P*-value of 0.83, indicating minimal distinction between the distributions, supporting the hypothesis of akin drives for L1 and L3 in the case of backward propagations.

The relevant distinctions and similarities between L1 and L3 spontaneous activity generation are more clearly evidenced through heat-maps (Figures 8C–F). Such type of plots provides relevant insight into the directional flow and distribution of spontaneous activity across segments. As previously mentioned, forward wave propagation reveals striking differences between L1 and L3 (Figures 8C, E). In L3, forward activity is highly concentrated at A8 and evolves consistently through most of the larva's body. In contrast, L1 displays a concentration of forward activity generation predominantly at A1 and T3, highlighting an important difference regarding the initiation vertices of contraction waves for the L1 and L3 individuals. Nonetheless, backward propagating waves for L1 and L3 exhibit fairly comparable patterns (Figures 8D, E).

### 3.2 Propagation of activity

**Figure 9:**
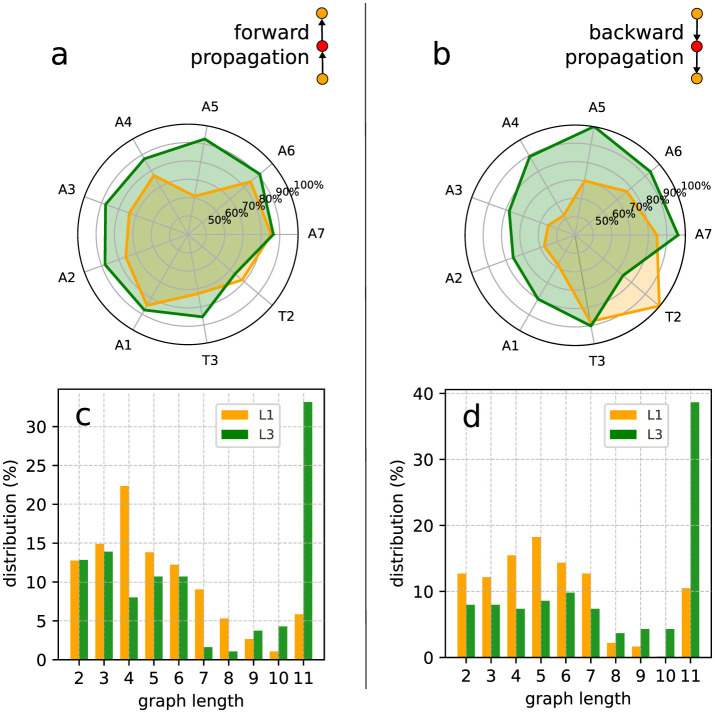
Inner excited activity per segment (so A8 and T1 are not included) of L1 and L3 larvae. The probability for a segment to continue the **(A)** forward and the **(B)** backward propagation once excited. The insets show a typical link (i.e., a very basic motif in the graph representation) of the propagation chain. The graph length (main text) distribution for *G*_*j*_'s representing **(C)** forward and **(D)** backward propagation.

Differently from spontaneous, “excited” events relate to activity triggered by preceding—but not later than Δτ—spikes in neighboring segment ROIs. In our construction, these events are represented by vertices attached to oriented edges. When these edges income from the posterior (anterior) region, inset in (Figures 9A, B), we have a forward (backward) propagating wave. Further, if these *v*_*i*_'s are also tied to outgoing oriented edges ${\vec{e}}_{ii^{\prime }}$, thus directed toward other vertices $v_{i^{\prime }}$'s, they form the interior links of a propagation chain, or equivalent, “inner” vertices of a graph *G*_*j*_. Obviously, the first vertex of any *G*_*j*_ is spontaneous, whereas the last is excited, but not an inner vertex. We denote as $V_{\text{inner}}^{L}\left(G_{j}\right)$ ($V_{\text{inner}}^{R}\left(G_{j}\right)$) the number of inner vertices of *G*_*j*_ related to the left (right) segments. The length of *G*_*j*_ is then assumed as the largest between $V_{\text{inner}}^{L}\left(G_{j}\right)$ and $V_{\text{inner}}^{R}\left(G_{j}\right)$. Such definition is useful if the interest is to characterize the neural activity just in terms of type of segments, regardless their locations.

Given a graph we can count the number of its vertices corresponding to a certain segment (A1 to A8 and T1 to T3). So, considering all the non-trivial *J*-*J*_1_ graphs obtained from the data, we can determine the relative contribution of any segment to the neural activity processes along a full experimental run. For instance, in Figures 9A, B we depict the probability of each inner segment to participate in, respectively, forward and backward waves. As one can see, with the exception of T2, all other subsequent segments for the L3 larvae are more likely to continue a propagating wave, either forward or backward.

From the comparatively lower propagation probability in the L1 larvae, one can infer that their neural signals present a higher tendency to fade away while traveling along the VNC, resulting in shorter activity chains, i.e., smaller *G*_*j*_'s. This can be quantified by computing the distribution of the previously defined graph length for *G*_*j*_'s representing forward and backward waves. This is displayed in Figures 9C, D. Note that for L3 larvae, both cases display peaks at eleven vertices (or segments), indicating activity across the entire neural system. On the other hand, for the L1 larvae the distributions maximum are at four (forward) and five (backward), confirming a propensity of wave abbreviation. In Table 2 we show the averaged probability that once excited, a segment will also stimulate another segment (so, propagating the signal). We see that the probabilities are always greater for L3 than for L1 and that backward waves are less likely of getting through than the forward waves.

**Table 2:** Probability (in %) of a segment, once excited, to also excite a next one during forward and backward wave propagation, averaged over each larvae population.

**Direction**	**Mean % L1 (SD)**	**Mean % L3 (SD)**	**MNE L1 (SD)**	**MNE L3 (SD)**
Forward	69.60 (5.60) %	77.45 (7.21) %	19.1 (8.62)	56.1 (47.65)
Backward	40.61 (12.33) %	64.62 (16.51) %	22.3 (12.28)	56.0 (41.56)

The mean number of observed propagation events (MNE) is also shown.

Finally, we applied the KS test to compare the forward and backward distributions (Figures 9C, D) for L1 and L3 larvae populations. For the forward of L1 and L3, the result was 0.2 with a *P*-value of 0.994, pointing to a no sensible difference between potential origins for the distributions. Conversely, for the backward of L1 and L3, the KS value was 0.6 with a *P*-value of 0.052, suggesting eventual distinct drives for L1 and L3. These findings might seem in contradiction with the same analysis done for the distributions in Figures 8A, B. However, although similar, the quantities in Figures 8, 9 represent complementary traits of the neural activity evolution. This key aspect will be addressed in Section 4.

### 3.3 The spatial features of neural activity

The exact succession structure of segments activation (resulting from underlying neuromotor processes) is paramount in generating the distinct functional movements in *Drosophila* larvae. For example, it is well established that sequential and symmetrical excitation of segments along both sides of the VNC yield forward and backward crawling (Loveless et al., 2019). In contrast, asymmetrical activity, i.e., when segments are prompted only on one side of the VNC, tends to control turning maneuvrers, a crucial component of larval navigation that shapes individuals trajectories (Pulver et al., 2015).

**Figure 10:**
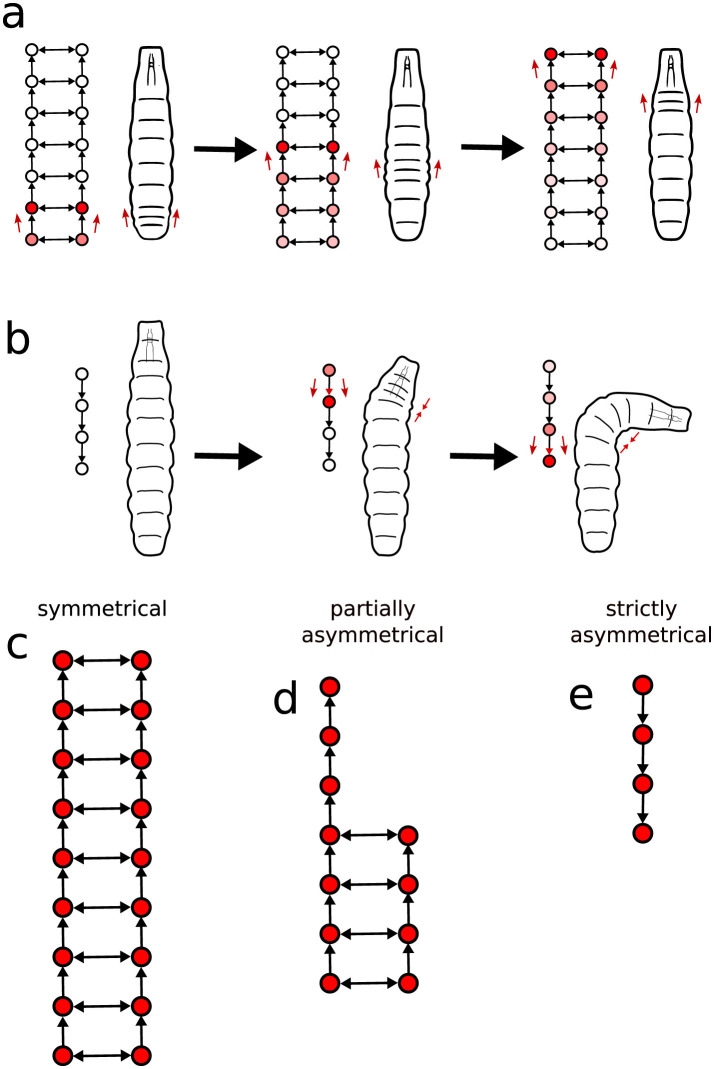
The spatial symmetry characteristics of the graphs constructed from the neural signals and relations to larvae basic movement steps. **(A)** Forward and backward crawling locomotion is related to neural activity propagation in the both sides of the VNC, whereas **(B)** body turns are usually associated to spikes propagating in just on side of the ganglia segments. Consequently, the graphs can be classified as either **(C)** totally symmetrical, **(D)** partially symmetrical, or **(E)** strictly asymmetrical.

To gain quantitative insight into the interplay between neural activity and movement patterns, we apply the present framework to better classify the neural signal waves in terms of their symmetry features. Specifically, we examine the symmetry edges (as defined in Section 2.6), which represent nearly simultaneous activation of a segment's left and right sides in the VNC. If the excitation of a segment is connected by a symmetry edge, we assume such segment to be symmetrically activated. Conversely, when the spike occurs solely on one side, the segment is considered asymmetrically activated. These situations are schematically illustrated in Figures 10A, B.

As it becomes clear by analyzing the obtained graphs, a propagating wave typically does not keep changing back and forth between symmetrical and asymmetrical motifs. Instead, it shows a distinct region of either symmetrical or asymmetrical activation, switching from one mode to the other at most just once along the way. Therefore, we can divide activity propagation into three cases.

1. *Symmetrical*: The engaged segments in a signal activate both the left and right sides of the VNC (Figure 10C).
2. *Partially asymmetrical*: Part of the activity wave propagates along both sides and part along a single side (Figure 10D);
3. *Strictly asymmetrical*: The propagation takes place only one side (Figure 10E).

In terms of actual larvae locomotion, the symmetrical graphs (associated to symmetrical VNC activity), relate to forward and backward crawling, once this type of displacement is generally correlated to bilateral segment contraction (Figure 10A). In contrast, both partially and strictly asymmetrical graphs are plausibly linked to turning behavior, reflecting the one-sided segment activation characteristic of directional changes (Figure 10B).

**Figure 11:**
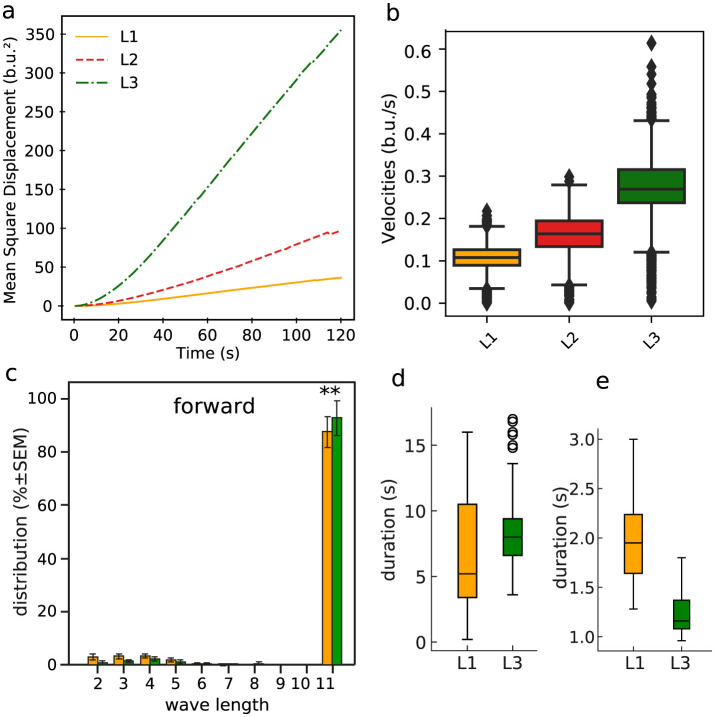
Some relevant statistical features of the larvae (straight) movement characteristics. **(A)** Normalized (by the body size) MSD. **(B)** Normalized velocities. **(C)** Distribution of the number of body segments (wavelengths) contracting during locomotion (ANOVA *F*_(17, 207)_ = 899, *R*^2^ = 0.99, *P* < 0.0001, Bonferroni *post-hoc* test for each length, ** means *P* < 0.01). **(D)** Time duration boxplot for the full size graphs of neuromotor activity (i.e., of length 11, thus all ROIs); L1 mean: 6.80 s, L3 mean: 8.31 s, KS = 0.47, *P* < 0.0001. **(E)** Time duration boxplot for the full forward locomotor waves (i.e., involving 11 body segments, so related to locomotion behavior); L1 mean: 1.97 s, L3 mean: 1.19 s, KS = 0.875, *P* < 0.001.

Motivated by the previous observations, we contrasted the symmetric graph structures for the L1 and L3 populations with average movement properties of the larvae in the first, second and third stage of their development (L1, L2, and L3). So, considering the full collection of trajectories, we show in Figure 11A the mean square displacement (MSD)—normalized by the body length—in each of the mentioned three consecutive stages. Clearly, the diffusion rate (given by the slop of the MSD) increases as the population maturates, i.e., evolving from day 1 to day 3 or equivalently from L1 to L3, with a particularly considerable greater diffusivity enhancement for L3. We hypothesized that behaviorally, shorter neural activity waves, frequent in L1, could translate into muscles contraction waves that might not travel the full length of the larvae's body. This should reduce the ability of propelling the larvae (either forward or backward) as effectively as when crawling waves transverse the full extend of the larvae's body. To test such supposition, we analyzed the waves of segment contraction in L1 and L3 crawling larvae. We depict in Figure 11C the resulting distribution of the number of body segments (so wavelength, as explained in Section 2.3) contracting during straight forward locomotion. The majority of the peristaltic waves propagate along the entire body in both L1 and L3 larvae. Shorter waves are infrequent and stereotyped, occurring in the anterior half of the body and helping to realign the front of the larva after a sharp turn. Further, short aborted waves, starting in A8 and advancing two segments, are very rare (Figure 11C). Therefore, there seems to have little relationship between the shorter wavelengths described in Figure 8 and the behavior of the larvae in more persistent straight locomotion, where proprioceptive inputs are likely to guarantee the correct propagation of peristaltic waves of neuromotor activity (Hughes and Thomas, 2007). We also investigated the distribution of time durations of full graphs (so, of length 11) of neural activity and full locomotor waves (so, comprising 11 body segments), respectively, in Figures 11D, E. Conceivably, their variability could be responsible for differences in crawling speed and dispersion. The average time duration of full graphs is longer for L3 than for L1. This probably reflects the dramatic increase in size of the VNC. On the other hand, this trend is reversed for the average time for a complete forward peristaltic wave (A8–T1), now with L3 faster than L1 (Figure 11E). In addition, there were clear differences in the number of forward waves executed per minute: 32.9 ± 2.2 w/min for L1 and 50.26 ± 3.5 w/min for L3 (*P* < 0.0001). These facts support the idea that developmental changes in larval speed are not solely dependent on changes in the VNC, but relies on the interaction with the muscles and proprioceptive inputs. For example, the shorter times needed to execute a full forward locomotor contraction wave for L3 may point to a more efficient control of the basic movement steps, even considering that the full neural signals of L3 tend to be longer.

**Figure 12:**
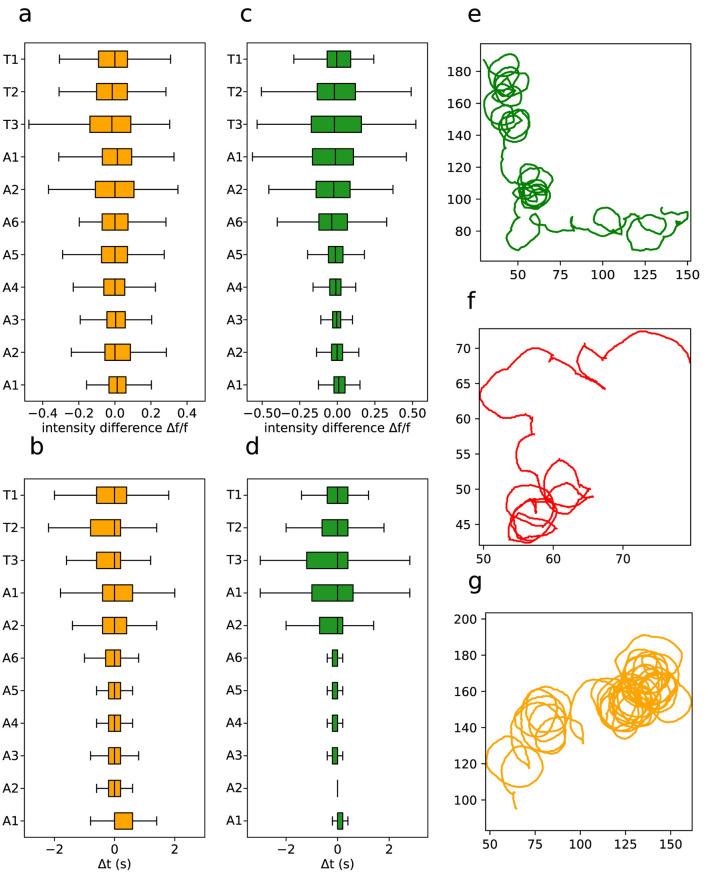
Asymmetries in neural waves and in movement patterns. **(A)** Intensity difference for neuronal activity per segment in L1 larvae. **(B)** Activity phase per segment in L1 larvae. **(C)** Intensity difference for neuronal activity per segment in L3 larvae. **(D)** Activity phase per segment in L3 larvae. Representative example of a **(E)** L1, **(F)** L2 and **(G)** L3 larvae curved trajectory. Note that each larva has a tendency to turn more in one direction, but as a full population there is little handedness if any (Wosniack et al., 2022).

We furthermore quantified phase and intensity differences between the left and right sides of segment activity. Figures 12A, C displays the intensity differences across segments, while Figures 12B, D depicts the phase differences. They are much more pronounced in anterior segments, for both L1 and L3 larvae. On the other hand, the distributions are similar for forward and backward propagating graphs, meaning that while backward waves initially exhibit distinct phase and intensity mismatches between the left and right sides, these discrepancies tend to dissipate as the wave propagates. This indicates a kind of activity synchronization as the neural signal propagates and that the phases and intensity differences are an inherent property of the segmental ROIs.

Actually, the observation that phase and intensity differences between sides are concentrate in the anterior segments might point to directional navigation mechanisms, once these are the ones that tend to control the movement direction in living larvae. In fact, a possibility is that they should cause in a biased impulse propelling the larvae's body forward, as one side of each segment would contract more than the other (at different times). Notably, there seems to be a directional bias on phase and intensity toward the left side of the VNC. A consistently non-symmetric pattern of crawling activity would result in a certain degree of circularity into the larvae's locomotion, which indeed was observed in the trajectory recordings, as shown in Figures 12E–G. Although it has been proposed that circular trajectory patterns in animals can arise from asymmetries in body structure (Sadeghi et al., 2000), the data suggest that these patterns could instead be driven by asymmetries in neurological activity itself.

**Figure 13:**
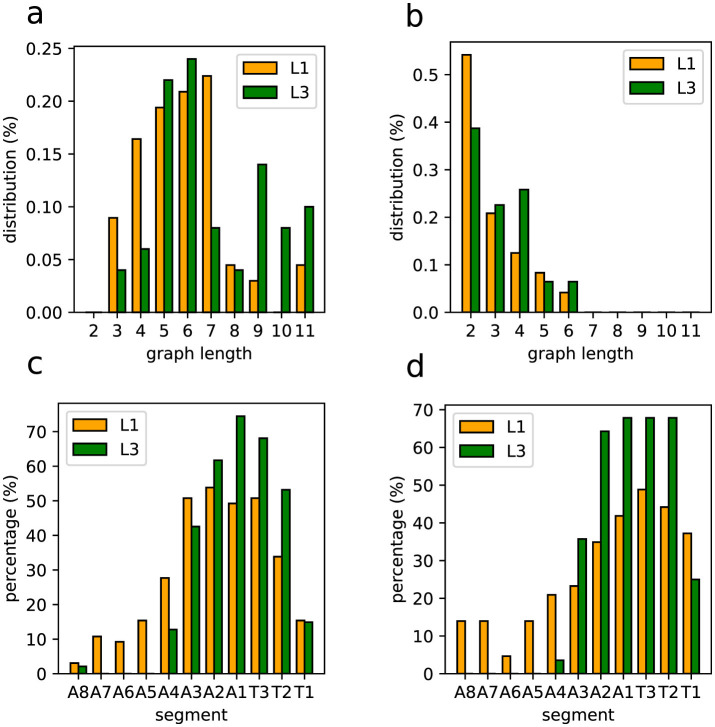
Statistical distributions of asymmetric graphs (hence related to unbalanced segments sides). **(A)** Graph length distribution for partially asymmetrical graphs. **(B)** Graph length distribution for strictly asymmetrical graphs. **(C)** Segment distribution of asymmetries in partially asymmetrical graphs. **(D)** Segment distribution of asymmetries in strictly asymmetrical graphs.

The lengths of asymmetric (partial or strict) neural activity graphs are shown in Figures 13A, B. It is relevant that strictly asymmetric activity appears only near the thoracic segments. Also, even in partially asymmetrical waves, the asymmetrical portions also tend to concentrate in this anterior region. So, as illustrated in Figures 13C, D, asymmetry is predominantly localized in the front segments of the VNC, thus agreeing with the expectation that turning movements primarily involve anterior body regions.

### 3.4 Developmental changes in individual neurons

**Figure 14:**
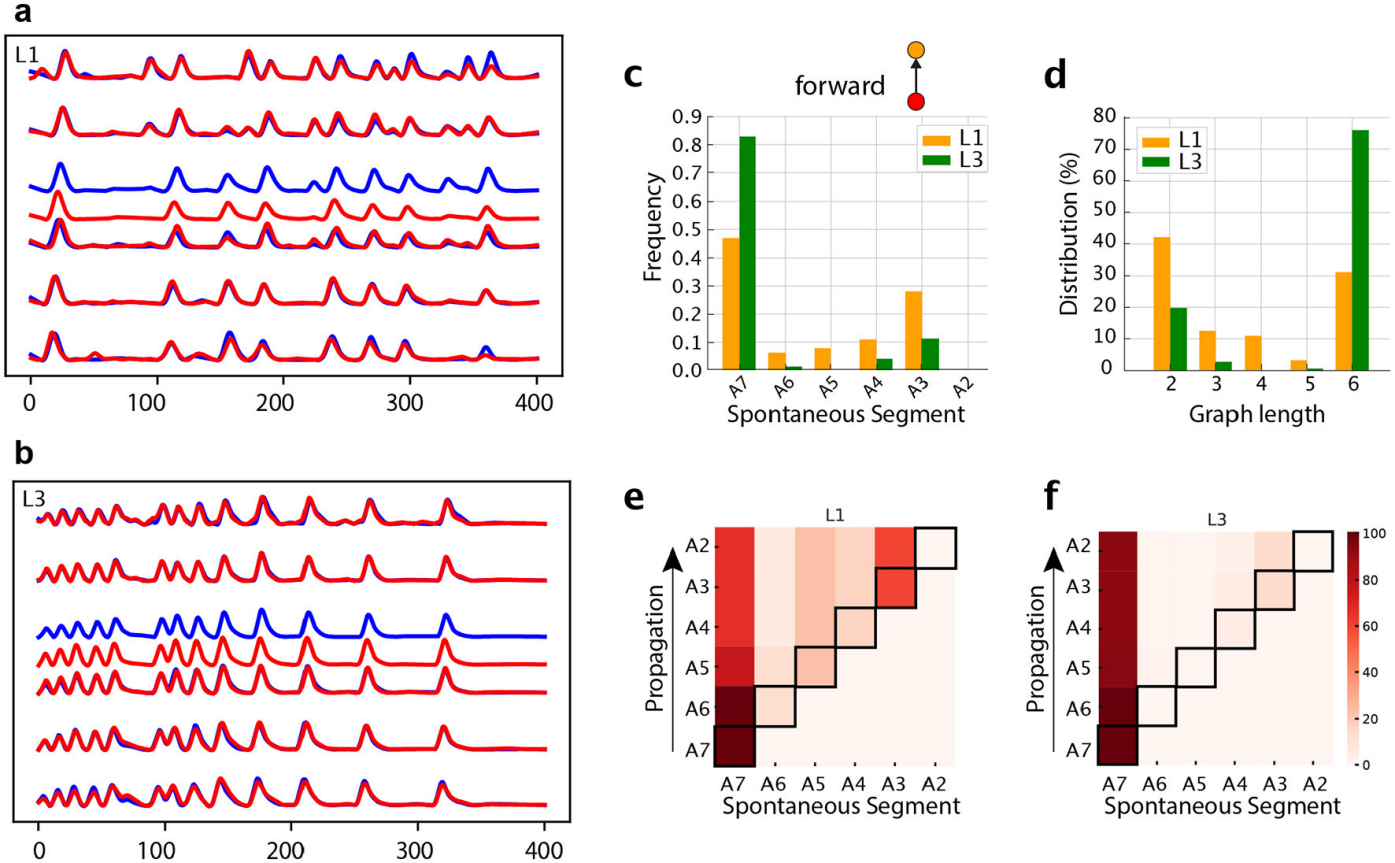
Analysis of a central pattern generator neuron, A27h. Representative calcium imaging traces of **(A)** L1 and **(B)** L3 VNCs. **(C)** Distributions for segments initiating forward waves. **(D)** Graph length distribution for *G*_*j*_'s representing forward propagation. **(E, F)** Heat maps in which the horizontal axes marks the origin of the activity chains, thus corresponding to the locations of the spontaneous segments (or vertices). The propagation occurs along each row (as shown by arrows). The color intensity indicates how frequently a given segment contributes to the associated propagation chain.

Having employed our graph construction approach to describe the characteristics of neuromotor activity, here we shall test it on an identified interneuron. We chose A27h, a premotor neuron thought to be part of the central pattern generator for crawling (Fushiki et al., 2016). A27h is particularly relevant to our study as it receives proprioceptive inputs that are necessary for the gap-junction coupling with M neurons and for the maturation of the crawling central pattern generator (Zeng et al., 2021). We analyzed A27h forward activity at the soma level from A2 to A7, Figures 14A, B. Our analysis of the activity patterns, comparing L1 with L3, shared some trends observed for the neuromotor activity. In L1 many waves are initiated in distinct segments along the VNC, while in L3 they are concentrated in A7 (Figure 14C). The distinct starting point of forward waves induces more distributed variable graph lengths (Figure 14D). Once waves are initiated, they tend to progress along the entire ventral nerve cord (Figures 14E, F). Therefore, our graph model has revealed that the disorganized spontaneous activity we observed in motorneurons in L1 is also present in certain central neurons, like A27h. The intersegmental propagation is very little affected in A27h (Table 3). This suggests that connectivity of other central pattern generator interneurons, like GDL (Fushiki et al., 2016) must be responsible for the defect in propagation observed at the neuromotor level in L1. In a future work, the present method will be used to reveal the interneurons involved.

**Table 3:** Probability (in %) of a segmental A27h neuron, once excited, to also excite a next one during forward wave propagation, averaged over each populations.

	**Mean % (SD)**	**MNE (SD)**
L1	92.48 (4.99)	33.80 (17.14)
L3	98.24 (1.99)	71.62 (21.35)

SD is the standard deviation, *T* test, *P* = 0.008. The mean number of observed propagation events (MNE) is also shown.

## 4 Discussion and concluding remarks

In this study, we explored the developmental changes in neuromotor activity patterns of *Drosophila melanogaster* larvae by employing calcium imaging techniques alongside a novel mathematical framework grounded in graph theory. By comparing first instar (L1) and early third instar (L3) larvae, we addressed how neural activity propagation correlates with locomotor behavior during development.

Our findings revealed significant differences between L1 and L3 larvae in terms of neural activity initiation and propagation. For instance, L1 larvae exhibited a higher frequency of spontaneous neural activity events that ended up failing to trigger successful activity chains. The starting points of these “broken” signals were fairly uniformly distributed across the ventral nerve cord (VNC). This is reminiscent of the developmental patterns of muscle contraction during the embryonic stage, when bursts of activity, displaying motifs resembling forward or backward crawling, begins at any segment. Therefore, the patterns of neuromotor activity we recorded in L1 suggest a less developmentally mature and coordinated neuromotor network (Pereanu et al., 2007)

Conversely, L3 larvae displayed spontaneous activity concentrated in specific segments associated with the initiation of forward and backward neural waves, and indication of a more developed and organized neuromotor system. Actually, propagation of neural activity was more efficient in L3 than in L1, with higher probabilities of successful forward and backward propagation across most segmental ROIs. However, there is little evidence that this fact might lead to higher larval dispersion in L3 than in L1, even though during crawling, most forward peristaltic waves start in A8, propagating all the way until reaching the thorax. Rather, these results point toward a role of proprioception for the uninterrupted propagation of neuromotor activity. Proprioception is known to play a role for the coordination of activity in adjacent legs or along the body segments (Hess and Büschges, 1999; Wen et al., 2018; Suster and Bate, 2002; Hughes and Thomas, 2007). In L1, isolating the nervous system from the body and its sensory inputs dramatically affects the ability of waves to successfully propagate, therefore suggesting that the intersegmental connectivity required for propagation has not yet completely matured. It has been shown that during larval development the size, number of terminal dendritic branches, and total number of synaptic inputs increases many folds while preserving cell type-specific connectivity (Gerhard et al., 2017). It is possible that the strength of intersegmental connections is still not sufficient to support the intersegmental propagation of neuronal activity.

The A27h results indicate that not all interneurons that are part of the central pattern generator (Fushiki et al., 2016) have seemingly immature connections in L1. Notably, A27h which, in coordination with the M neuron, requires early proprioception inputs for the development of the larval central pattern generator for locomotion (Zeng et al., 2021), is mature enough to induce mostly successful propagation of activity, even when the overall neuromotor activity produces truncated waves. In L3, the intersegmental propagation must rely on central connectivity in the VNC, while proprioception controls other aspects as speed, intensity of muscle contraction and maneuverability (Hughes and Thomas, 2007; Pulver et al., 2015; Gebehart and Büschges, 2024).

The increase in locomotor dispersion observed in older larvae is likely to be related to the speed of activity propagation along the entire VNC in the intact animal. In fact, from a behavioral point of view, there is an ~65% rise in the speed of the peristatic wave of segments contraction along the whole body from L1 to L3. We also identified a growing frequency in the number of forward segment peristaltic waves generated per minute. Such waves often start before the ending of the previous one. Furthermore, it was shown that the increased size of the larvae makes each step bigger, leading to longer displacements (Almeida-Carvalho et al., 2017). In comparison, in the isolated nervous system (i.e., in dissected larvae), the time duration of full neuromotor activity waves (see Figures 11D, E) are longer in older L3 larvae, probably as a consequence of the greater length of the VNC. This latter apparent contradiction reveals the importance of the body proprioception and muscle development for change in speed of the actual larvae. Physiologically, at the neuromuscular junctions, motoneurons enlarge their presynaptic axon terminals in size and strength (Schuster et al., 1996) and increase glutamate release (Rasse et al., 2005). While within the central nervous system, motoneurons enlarge their postsynaptic dendritic arbors inducing synaptic strengthening that enhances neuronal activity (Zwart et al., 2013). These mechanisms guarantee the effective contraction in the remarkably bigger L3 muscle, and may as well alter the speed and strength of fibers contraction necessary for speed magnification.

During symmetrical activity, we observed phase and intensity differences between the left and right sides of the VNC, particularly in the anterior segments. These may contribute to the curved trajectories commonly seen in larval movement (Gomez-Marin and Louis, 2014; Almeida-Carvalho et al., 2017). Further, such differences suggest that neural activity patterns, rather than structural asymmetries in the body (Sadeghi et al., 2000), might underlie certain locomotor behavior like turning and circular movement patterns (Souman et al., 2009). By its turn, asymmetrical neural activity was predominantly observed in anterior segments and is probably associated with turning behaviors, enabling larvae to better navigate through the environment. Also, the concentration of asymmetrical activity in these segments points to developmental maturation which enhances directional changes and maneuverability.

Lastly, from a broader point of view, by introducing a quantitative graph-based model to represent neural activity propagation, the present work provided a systematic framework to analyze and compare neuromotor patterns across developmental stages. For example, it allowed to identify specific motifs and propagation probabilities, offering insights into the evolvement of neural circuits responsible for locomotion. Potentially, the present methodology can be useful in the concrete characterization of distinct spontaneous patterns of neuronal activity. This includes neurons that are central, as the here discussed A27h, and others that generate distinct “tasks,” like: rolling (Cooney et al., 2023), self-righting (Picao-Osorio et al., 2015), hunching (Francis et al., 2024), etc. It also could help to typify the consequences of precise neuronal manipulations affecting speed (Kohsaka et al., 2014; Hiramoto et al., 2021), left right coordination (Heckscher et al., 2015) or more generally any changes in regionally coordinated neuronal activity (Masson et al., 2020; Gerhard et al., 2017).

Future research could employ our mathematical formalism to model patterns of segment contraction during movement, trying to correlate them with neuronal activity. So, we believe this method provides a more generic perspective of how neural networks form and organize to create coordinated behaviors. This should have consequences not only for developmental biology but also for understanding motor control and neural coordination in more complex organisms.

## Data Availability

The raw data supporting the conclusions of this article will be made available by the authors, without undue reservation.
